# Astrovirus Infection in Hospitalized Infants with Severe Combined Immunodeficiency after Allogeneic Hematopoietic Stem Cell Transplantation

**DOI:** 10.1371/journal.pone.0027483

**Published:** 2011-11-11

**Authors:** Werner Wunderli, Astrid Meerbach, Tayfun Guengoer, Christoph Berger, Oliver Greiner, Rosmarie Caduff, Alexandra Trkola, Walter Bossart, Daniel Gerlach, Manuel Schibler, Samuel Cordey, Thomas Alexander McKee, Sandra Van Belle, Laurent Kaiser, Caroline Tapparel

**Affiliations:** 1 Division of Clinical Virology, University of Zurich, Zurich, Switzerland; 2 Laboratory of Virology, Division of Infectious Diseases and Division of Laboratory Medicine, University of Geneva Hospitals, Geneva, Switzerland; 3 Division of Immunology and Bone Marrow Transplantation, University Children's Hospital, Zurich, Switzerland; 4 Division of Pathology, University of Zurich Hospitals, Zurich, Switzerland; 5 Department of Genetic Medicine and Development and Swiss Institute of Bioinformatics, University of Geneva Medical School, Geneva, Switzerland; 6 Swiss National Reference Centre for Emerging Viruses (CRIVE), University of Geneva Hospitals, Geneva, Switzerland; 7 Division of Pathology, University of Geneva Hospitals, Geneva, Switzerland; University of California, San Francisco, United States of America

## Abstract

Infants with severe primary combined immunodeficiency (SCID) and children post-allogeneic hematopoietic stem cell transplantation (HSCT) are extremely susceptible to unusual infections. The lack of generic tools to detect disease-causing viruses among more than 200 potential human viral pathogens represents a major challenge to clinicians and virologists. We investigated retrospectively the causes of a fatal disseminated viral infection with meningoencephalitis in an infant with gamma C-SCID and of chronic gastroenteritis in 2 other infants admitted for HSCT during the same time period. Analysis was undertaken by combining cell culture, electron microscopy and sequence-independent single primer amplification (SISPA) techniques**.** Caco-2 cells inoculated with fecal samples developed a cytopathic effect and non-enveloped viral particles in infected cells were detected by electron microscopy. SISPA led to the identification of astrovirus as the pathogen. Both sequencing of the capsid gene and the pattern of infection suggested nosocomial transmission from a chronically excreting index case to 2 other patients leading to fatal infection in 1 and to transient disease in the others. Virus-specific, real-time reverse transcription polymerase chain reaction was then performed on different stored samples to assess the extent of infection. Infection was associated with viremia in 2 cases and contributed to death in 1. At autopsy, viral RNA was detected in the brain and different other organs, while immunochemistry confirmed infection of gastrointestinal tissues. This report illustrates the usefulness of the combined use of classical virology procedures and modern molecular tools for the diagnosis of unexpected infections. It illustrates that astrovirus has the potential to cause severe disseminated lethal infection in highly immunocompromised pediatric patients.

## Introduction

Infants with severe combined immunodeficiency (SCID) and children after allogeneic hematopoietic stem cell transplantation (HSCT) are exceptionally susceptible to viral infections and viral reactivations. The lack of functional cytotoxic T- and NK-cells prior to and for a certain time after HSCT opens the door to infections by unexpected pathogens either community acquired or nosocomial. Viral infections, including those that commonly cause self-limited childhood diseases, can lead to protracted infections with chronic viral shedding, but also to disseminated disease with infection of organs rarely affected in immunocompetent hosts [1]. When highly immunocompromised infants present prolonged illness despite broad spectrum antimicrobial therapy, extended microbiological investigations should be considered. Routine viral screening is limited to the most frequent groups of viruses including herpes-, hepatitis-, respiratory-, adeno-, polyoma- and selected gastrointestinal viruses. However, the number of different viruses potentially pathogenic in humans is estimated to be more than 200 [2]. Therefore, when standard investigations remain negative despite clinical suspicion for viral disease, screening has to be extended and depends on the availability of in-house assays. Under certain circumstances research techniques should be considered. Unfortunately, the clinical features presented by transplanted infants or patients with SCID are not always typical and often misleading. Generic molecular tools, such as microarrays [3], ultra-deep sequencing [4], sequence-independent single primer amplification (SISPA) [5], virus discovery based on *c*DNA-amplified fragment length polymorphism [6], or any other similar procedures, offer potentially attractive alternatives although the sensitivity is limited.

We describe here the retrospective analysis of a cluster of 3 infants prior to or after allogeneic HCST infected with an initially unrecognized enteric virus. It was first detected by cell culture of fecal specimens and identified as an astrovirus using a modified SISPA protocol. Subsequent screening with a specific real-time reverse transcription polymerase chain reaction (RT-PCR) assay of different patients from the same time period revealed a cluster of 3 cases that remained undetected by standard investigation. In 1 fatal case, the infection involved multiple organs, including the central nervous system. Viral genome sequencing revealed that all cases were infected with the same astrovirus type 4 strain.

## Materials and Methods

### Cultivation of astrovirus, preparation for electron microscopy (EM), and immunofluorescence (IF)

Caco-2 cells (ATCC # HTB 37) were cultivated in M199 medium containing 10% fetal calf serum, glutamine and a mixture of penicillin/streptomycin. Confluent cells in culture tubes were used for the inoculation of specimens. Stool samples were suspended in PBS (10% v/v) and centrifuged at 2000 g for 20 min. The supernatant was then removed and penicillin, streptomycin, and fungizone were added. Culture tubes were inoculated with 200 µl of the suspension in 2 ml culture medium without the addition of trypsin (Tables S1,S2,S3). Cultures were screened periodically for cytophathic changes for up 10 days and compared with a non-infected cell-culture control. If rounding up of cells started to appear (usually after 24–72 h), they were scraped off and used to prepare cytospin slides for IF staining. After drying and fixation of the slides, cells were incubated with monoclonal antibodies directed against enteroviruses (pan-enterovirus blend, Chemicon International). Of note, the antigen specificity of these antibodies is not specified by the company and potential cross-reaction with hepatitis A, reovirus 3, and some rhinovirus and astrovirus strains is indicated in the data sheet. Some samples (Tables S1,S2,S3) were analyzed retrospectively by IF after Caco-2 cell infection with the pan-enterovirus blend kit and, in parallel, with anti-astrovirus-specific monoclonal antibody (Argene 11-301) diluted 1/100 in PBS^--^-1% BSA before addition of the anti-mouse IgG AB/FITC (Light Diagnostics).

For EM, Caco-2 cells were infected with a 10% stool suspension of a positive sample from patient 1 (sample no.12869; Table S1). After adsorption for 2 h, the medium was changed and cells were further incubated at 37°C with 5% CO2 for 2 days. Cells were removed by trypsination and centrifuged at 900 g for 5 min. The supernatant was removed and the cell pellet was fixed in glutaraldehyde. After dehydration, the cell pellet was embedded in epoxy resin and thin cuts were processed for standard EM investigation. This procedure was performed by the Central EM Service Unit of the University of Zurich.

### Screening of patient material for the presence of infectious agents

Written informed consent was obtained from the children's parents and covered all investigations on patient samples. The study was approved by the institutional ethics committee of the University of Zurich Childreǹs Hospital, Zurich, Switzerland.

Standard histopathology was done on 5 µm-formalin-fixed, paraffin-embedded tissue sections stained with hematoxylin-eosin, Giemsa, and periodic acid Schiff (PAS). Additional staining (according to Brown-Brenn, Ziehl-Neelsen and Grocott) as well as immunohistochemistry for toxoplasmosis, herpes simplex virus (HSV), varicella zoster virus (VZV), cytomegalovirus (CMV), human herpes virus 8, parvovirus, and adenovirus (ADV) were conducted, including PCR for *mycobacterium* tuberculosis-complex and atypical nontuberculous mycobacteria.

Clinical specimens were screened for the presence of the following viruses by real-time PCR or real-time RT-PCR as previously described: influenza-, respiratory syncytial-, parainfluenza-, rhino-, entero-, metapneumo-, corona-, boca-, ADV, CMV, Epstein-Barr -, HSV 1 and 2, noro-, parvovirus B19, and VZV [7]–[14]. Only results from tests requested by the clinicians for the detection of viruses are summarized in Tables S1,S2,S3. Other tests were performed when the retrospective analysis on stored samples was done to exclude other infections. Screening for rotavirus was done by a latex agglutination kit (Orion Diagnostics, Finland).

### Identification of astrovirus and retrospective screening of stored specimen

Astrovirus cloning and sequencing were performed on inoculated infected Caco-2 cell supernatant (Table S1). For SISPA [5], RNA was extracted with TRIzol (Invitrogen) from 200 ul of infected Caco-2 cell supernatant and eluted in 20 ul of water. 9 ul of RNA were reverse transcribed with primer FR20RV-N (5′GCCGGAGCTCTGCAGATATNNNNNN3′) with Superscript II (Invitrogen) according to the manufacturer's instructions. The cDNA was then treated for 1h with 2.5 units of Klenow (New England Biolabs) before inactivation (10 min at 75°C). The resulting double-stranded DNA was then PCR-amplified with Taq Hifi polymerase (Invitrogen) with primer FR20RV (5′GCCGGAGCTCTGCAGATAT3′) according to the manufacturer's instructions. Amplification products were separated by electrophoresis on agarose gel and fragments (0.6–2.5 kb) were extracted with the QIAquick Gel Extraction kit (QIAGEN). Purified products were cloned using the TOPO TA cloning kit (Invitrogen). Minipreps were prepared and clones with the largest inserts were selected for sequencing with the ABI Prism 3130XL DNA Sequencer (Applied Biosystems). Chromatograms were imported for proofreading and assembly with the Geneious Pro 5.0.3 software (Biomatters Ltd). Blast analysis was done with http://blast.ncbi.nlm.nih.gov/Blast.cgi. Specific forward and reverse primers (astro4 FwdA: 5′CGTGCATCCTCCTTAATCC3′, astro4 FwdB: 5′ATCTTGAATCACTCCATGGG3′, astro4 RevA: 5′GAAGCATTATCATTTGTGTTTGTTAA3′ and Astro4 RevB: 5′CGGCCATTGTTATTGACC3′) were then designed to amplify and sequence the related regions of additional specimens collected from patients 1, 2 and 3 (Tables S1,S2,S3). Resulting sequences (GenBank accession # HQ396880 to HQ396890) were aligned with human astrovirus serotypes 1 to 8, as well as bat astrovirus sequences and cut to 543 nt. Alignments were constructed using MUSCLE [15] with default parameters. Multiple FastA was converted into PHYLIP format with the EMBOSS program seqret [16]. Trees were built with PhyML [17] using the GTR model, BIONJ for the initial tree, and optimized tree topology and branch lengths. The transition/transversion ratio was set to 4, and relative substitution rate categories were set to 16. The gamma shape parameter alpha was set to 1. The proportions of invariant sites were estimated from the data.

Astrovirus real-time RT-PCR was adapted from Logan et al [18] with 600nM of Hast.fwd, 900nM of HastV.rev, and 150nM Hast.Vpro (5′-FAM-CAACTCAGGAAACARG-MGB) and run under previously described experimental conditions [13] to retrospectively screen clinical samples and paraffin-embedded tissues. RNA was extracted with Easymag (Biomérieux) for clinical specimens, and with the Absolutely RNA(R) FFPE Kit (Stratagene) for paraffin-embedded autopsy samples. As an internal control, 10 µl of standardized canine distemper virus (CDV) of known concentration were added to each sample before extraction to monitor for the RNA isolation and amplification procedures.

Immunohistochemistry for astrovirus detection was performed on 3–5 µm autopsy tissue sections. The primary mouse anti-astrovirus monoclonal antibody (Argene 11-301) and the secondary antibody (Envision Flex, Dako, K 8010) were used at 1∶50 and undiluted, respectively. An irrelevant isotype IgG1 diluted 1∶10 was used as a negative control on the gut biopsy that was then revealed by the same secondary antibody. Anti-keratin II (Dako, M 3515) and anti-Glial Fibrillary Acidic Protein (GFAP) (Dako, Z 0334) antibodies were used as positive controls for small intestine and brain tissue staining, respectively. A senior pathologist, blinded to any viral real-time RT-PCR results, reviewed all slides.

## Results

### Identification of the infecting virus

**Figure 1:**
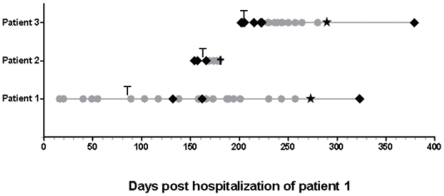
Timeline for the detection of astrovirus in samples of the 3 patients. Sample screening listed in Tables S1,S2,S3 is summarized as follows. Grey circles indicate positive detection of astrovirus in any type of samples (stools, serum, plasma, nasopharyngeal swabs, and vesicle swabs) by real-time RT-PCR and/or cell culture confirmed by immunofluorescence. Black diamonds indicate negative astrovirus detection by real-time RT-PCR. Black star represents the end of hospitalization. T: bone marrow transplantation; †: death.

**Figure 2:**
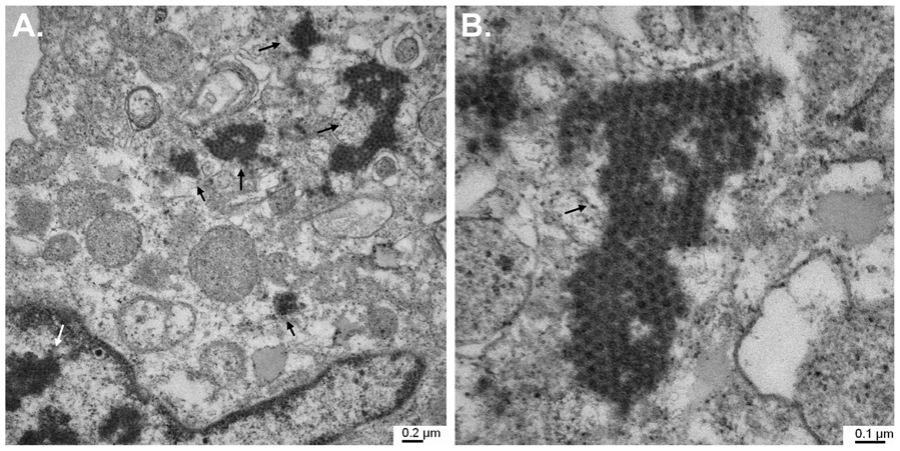
Electron microscopy of Caco-2 cells infected with a suspension of stool sample # 12869 from patient 1 (Table S1). A. 25,000-fold magnification of a portion of the infected Caco-2 cell containing both nucleus and cytoplasm. Dark staining in the nucleus (white arrow) is linked to chromatin condensation and granulation, whereas staining in the cytoplasm (black arrows) is due to aggregates of viral capsids. B. 66,000-fold magnification of viral capsid aggregates (black arrow) present in the cell cytoplasm.

**Figure 3:**
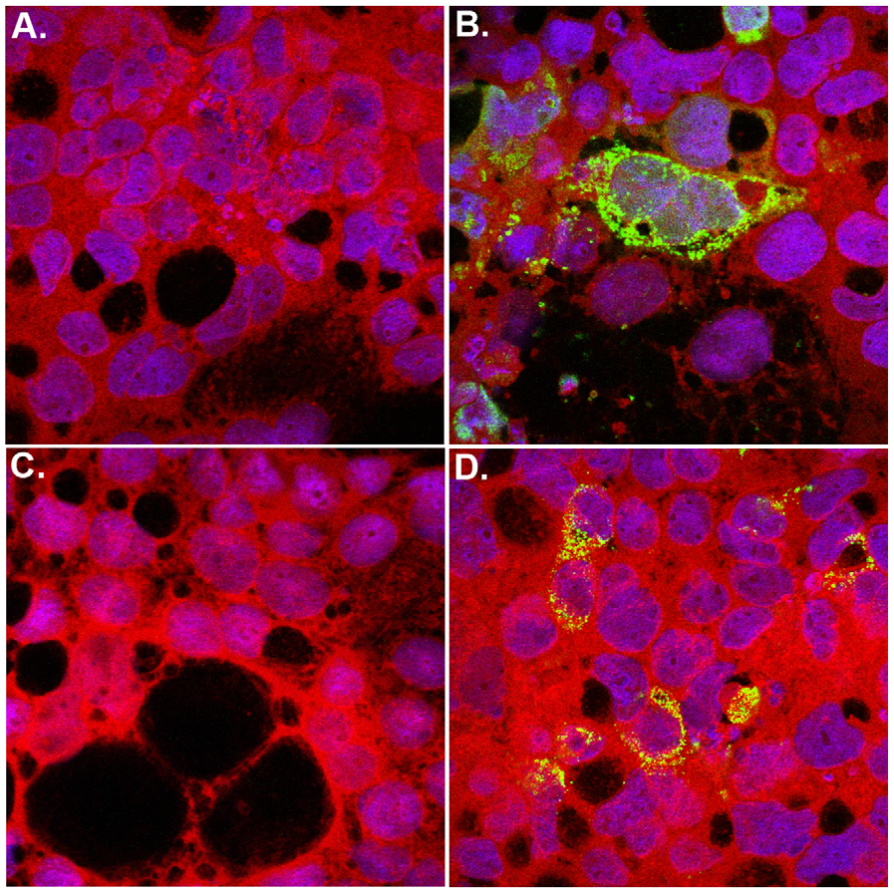
Immunofluorescence staining of non-infected (A,C) and infected (B,D) Caco-2 cells incubated with anti-astrovirus (A,B) and pan-enterovirus blend (C,D) monoclonal antibodies; magnification 63x (oil).

Three infants (patients 1-3) suffering from severe congenital immunodeficiency were admitted for allogeneic HSCT at the University of Zurich Children's Hospital during the same time period (Figure 1). Upon presentation of gastrointestinal symptoms with or without fever during hospitalization, body fluids and secretions were screened for the detection of the causative agent. None of these investigations allowed precise identification of the underlying disease (results not shown). However, a stool sample from patient 1 inoculated on Caco-2 cells showed an unexplained cytopathic effect (Tables S1) and prompted a closer investigation of routine samples from this ward. Stool samples from patients 1 and 2 gave a reproducible cytopathic effect, which consisted in rounding up and subsequent detachment of cells. A positive signal by IF was observed with the pan-enterovirus blend antibody (Chemicon International). Enteroviral real-time RT-PCR was negative (Tables S1,S2,S3) in all samples. According to the manufacturer's instructions, this antibody is known to cross-react with other viruses, such as hepatitis A, reovirus 3, astro- and rhinovirus. Cells from a positive culture from a stool sample from patient 1 were embedded and prepared for EM. Aggregates of typical spherical structures from non-enveloped viruses with icosahedral capsids of approximately 30 nm diameter (Figures 2A and 2B) were found in the cytoplasm of the infected cells. Application of the SISPA method allowed the identification of an astrovirus. 5 of 13 clones sequenced thanks to this method represented astroviral sequences (1 clone covered nt 2540 to 2948 of the reference astrovirus 4 genome [GenBank DQ344027]); the second, nt 3140 to 3795; and the 3 remaining clones overlapped over nt 4101 to 5094. The 8 remaining clones contained human sequences. IF performed with a specific anti-astrovirus monoclonal antibody retrospectively confirmed the cross-reactivity of the pan-enterovirus blend antibody with astrovirus-infected cells (Figure 3).

To investigate when and how the astrovirus infection was introduced, stored, frozen clinical samples of a total of 5 suspected cases were retrospectively screened. This was primarily done since patient 1 who had a T-B+NK-(gamma-C) SCID was hospitalized 5 months earlier than the deceased patient with chronic diarrhea and suspected viral disease. In fact, besides patient 1, 2 other patients were identified positive for astrovirus. Since patient 1 had stayed on the ward for a cumulative period of 9 months with ongoing gastrointestinal symptoms until he became T-cell engrafted, he was suspected to be the index case responsible for the other infections (Figure 1). The first sample, which retrospectively tested positive in patient 1, was from 11 July 2008 (Table S1), a few months before patients 2 and 3 were hospitalized (Tables S2 and S3).

### Clinical description of the 3 cases

#### Patient 1 (index case)

A 7-month-old boy was hospitalized in a reduced general condition with frequent stools, chronic cough, and fever. Following *pneumocystis jirovecii* pneumonia, he was diagnosed with an X-linked (gamma-C deficient) T-B+NK- form of SCID. At the age of 10 months (September 2008), he underwent allogeneic unrelated HLA-identical HSCT after pretreatment with rabbit anti-T-cell antibody (ATG) to prevent graft-versus host disease (GvHD), but without chemotherapeutic conditioning since he was critically ill. Despite laminar-airflow conditions, regular administration of intravenous immunoglobulins (virus inactivated by cold ethanol fractionation, solvent/detergent treatment, pH 4 inactivation, and ultra filtration; 0,4 g/kg every 2 weeks), and the strict use of masks and hand hygiene antisepsis measures, he developed a protracted respiratory parainfluenza virus type 3 infection, as well as a transient rhinovirus infection (Table S1), and was treated with oral ribavirin. At week 4 post- transplantation, persistent diarrhea increased and prompted a stool culture that revealed a positive cytopathic effect and was the index specimen used for astrovirus identification. Gastrointestinal symptoms deteriorated with bloody diarrhea without evidence for GvHD. Stool specimens collected before and after transplantation revealed retrospectively that the infection was acquired very early or even before hospitalization. Viral shedding persisted for almost 9 months until immune reconstitution (Figure 1; Table S1). Patient history revealed that the whole family, including patient 1, had suffered from a gastroenteritis infection of unknown cause in May 2008. Astrovirus RNA could also be detected in nasopharyngeal secretions and serum (Table 1). The patient was thus secreting large amounts of astrovirus when patients 2 and 3 were hospitalized in the same ward (Figure 1). Concomitant to T-cell immune reconstitution 4 months after HSCT, chronic diarrhea and respiratory symptoms stopped and both astrovirus and parainfluenza 3 infections disappeared.

**Table 1:** Detection of astrovirus in different samples from the 3 patients.

	Duration (in days) of astrovirus identification in different samples from the 3 patients		
Type of specimen	Patient 1	Patient 2	Patient 3
Stool	302	4	82
Serum	1	-	-
Plasma	-	8	-
Nasopharyngeal swab	71	-	1
Pharyngeal swab	167	-	1
Vesicle swab	-	1	-
Brain	-	PM	-
Heart	-	PM *	-
Lung	-	PM *	-
Spleen	-	PM *	-
Bone marrow	-	PM	-
Kidney	-	PM *	-
Small intestine	-	PM	-
**Duration of hospitalization**	**180**	**17**	**70**

-, not tested or negative; PM, post mortem autopsy tissue tested positive (CT values<36); PM*, post mortem autopsy tissue tested weakly positive (CT values > 36). See Tables S1,S2,S3 for detailed results.

#### Patient 2

**Figure 4:**
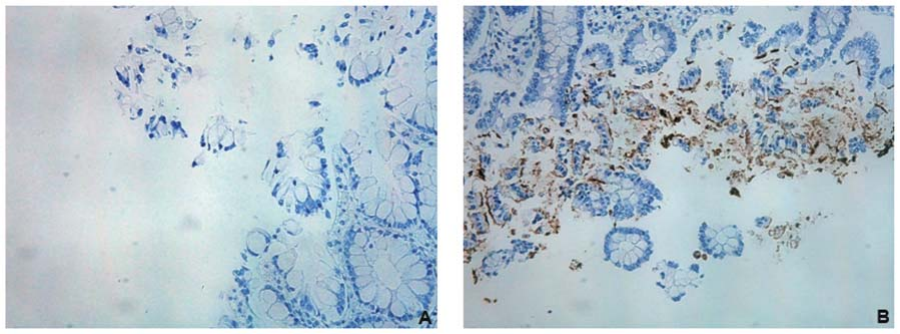
Immunohistochemistry of the small intestine autopsy tissue sections of patient 2. Immunohistochemistry with an anti-astrovirus antibody performed on non-infected (A) and infected (B) regions of the small intestine autopsy tissue sections.

A male infant and first-degree cousin of patient 1 was screened immediately after birth for SCID and was found to be also gamma C-deficient. Regular intravenous immunoglobulin infusions and cotrimoxazole and itraconazole prophylaxis were initiated and the patient remained at home until transplantation. An unrelated matched donor was identified and he received HSCT at the age of 3 months (5 December 2008). Since he had no clinical or laboratory hints for viral or other opportunistic infection prior to transplantation, he received conditioning with intravenous busulfan with therapeutic drug monitoring and rabbit ATG. Respiratory and intestinal virus screening cultures were negative prior to HSCT. Two days after infusion of the bone marrow, he developed high grade fever that remained persistent and of unknown origin, despite extensive microbiological and radiological investigations and broad spectrum antibiotic and antifungal treatment. Information from the donor center revealed that the donor had not suffered from fever or gastrointestinal disease prior to or following bone marrow sampling, thus excluding transmission by the graft itself. Cultures from two stool samples taken on 11 and 15 December revealed the same cytopathic effect observed in patient 1. However, no virus could be detected by standard screening procedures applied at that time, which did not include screening by the astrovirus-specific real-time RT-PCR (Table S2). Multiorgan dysfunction developed, including hepatic dysfunction and pulmonary infiltrates with respiratory distress and seizures, which required patient admission to the intensive care unit. Blood culture and other usual bacterial, mycobacterial, and fungal screening remained negative. Molecular screening for respiratory viruses in nasopharyngeal swabs was negative (Table S2) and none of the viruses tested could be detected in plasma. Cerebrospinal fluid real-time RT-PCR was negative for enterovirus. Cerebral magnetic resonance imaging revealed signs suggesting a severe meningoencephalitis and the patient died on day 17 post-transplantation. At autopsy, the heart, lungs, liver, bone marrow, spleen, and testicles showed a severe necrotizing inflammatory process. Neuropathological investigations revealed distinct inflammatory leptomeningeal and ventricular infiltrates consisting mainly of macrophages and granulocytes (Figure S1). In addition, fresh necrosis was detected in the hippocampus, basal ganglia, brain stem, and cerebellum. No infectious agent was found by special staining and immunohistochemistry. Retrospective screening of specimens by culture and/or real-time RT-PCR revealed that the first astrovirus-positive sample was collected at day 13 post-hospitalization (Figure 1; Table S2). Subsequent stool, plasma. and vesicle swab specimens revealed positive (Table 1). RNA was extracted from paraffin-embedded autopsy samples and tested by real-time RT-PCR. The small intestine was clearly astrovirus-positive (consistent with high viral loads [CT values of 22; Table S2]; bone marrow and brain were also positive, but with a lower viral load (CT around 30); kidney, spleen, lung and heart tissues were weakly positive (CT values over 36), whereas the liver was negative (Table 1; Table S2). Immunohistochemistry was performed on the same autopsy tissue after a preliminary validation on paraffin-embedded, astrovirus-infected Caco-2 cells (data not shown). The small intestine autopsy tissue section stained positive (Figure 4), but no signals were detected in the brain and bone marrow tissues although an anti-GFAP control antibody resulted in the expected positive signals (data not shown). Positive staining likely correlates with high viral load.

#### Patient 3

A 13-month-old boy with a profound T cell deficiency and hypogammaglobulinemia (presumed ZAP 70 defect) had suffered from several episodes of bacterial infections and *pneumocystis jirovecii* pneumonia. At the age of 16 months (January 2009), he underwent an allogeneic HSCT from an unrelated donor after reduced intensity conditioning. After 1 week, the patient developed gastrointestinal symptoms with recurrent diarrhea, nausea and vomiting without clinical evidence of GvHD. Fecal and respiratory specimens revealed retrospectively to be astrovirus-positive with the first positive sample detected 28 days post-hospitalization (Table 1; Figure 1; Table S3). No blood sample was available and viremia could not be documented. Gastrointestinal symptoms improved simultaneous to emerging T cells and moderate reduction of immunosuppression. The patient was discharged on day 70 in good general condition.

### Astrovirus transmission

It is very likely that patient 1 was the index case for the following reasons. 1) In May 2008, the patient's family reported an episode of virally transmitted gastrointestinal disease where patient 1 was also infected. We could not document this hypothesis as his severe immunodeficiency had not yet been detected at hospital admission and, therefore, no stool samples had been analyzed from the patient and his family. As he suffered from respiratory symptoms related to *pneumocystis jirovecii* pneumonia, his concomitant diarrhea was not further investigated. However, due to the absence of T- and NK-cells in gamma SCID, a successful elimination of his acquired viral gastrointestinal infection is not probable. 2) Stool samples were only tested by standard cell culture at the beginning of hospitalization. Because no cytopathic effect was observed, no IF test was done and samples were eliminated after 2 months. For this reason, no real-time RT-PCR could be done. The first strong positive sample was on day 23 (11 July 2008; Table S1) of hospitalization. Virus secretion in patient 1 could be documented on different samples until 9 March 2009. 3) He had been secreting large amounts of astrovirus (CT values: 15.3 and 12.23, respectively) when patients 2 and 3 were hospitalized in the same ward and was the very likely source of contamination for the 2 other cases (Figure 1).

When the 3 cases were hospitalized, standard precautions for hospitalized (according to US Centers for Disease Prevention and Control guidelines) and immunocompromised patients in the bone marrow transplantation (BMT) unit (e.g. care in single-patient, positive-pressure, laminar airflow boxes, introduction of disinfected material only, the wear of coats and masks for staff and parents entering the box) were applied. However, subsequent investigation by the infection control team revealed gaps and negligence in some aspects of standard precautions, such as handling and delivery of meals, hand hygiene of healthcare workers, and insufficient information provided to parents. This is of particular importance as the families of 2 patients were related. In addition, the BMT unit was overcrowded both inside and outside the boxes, thus preventing efficient environmental cleaning. In addition, disinfection studies showed that the products used for standard BMT procedures in our ward were insufficient in completely inactivating enteroviruses (data not shown).

**Figure 5:**
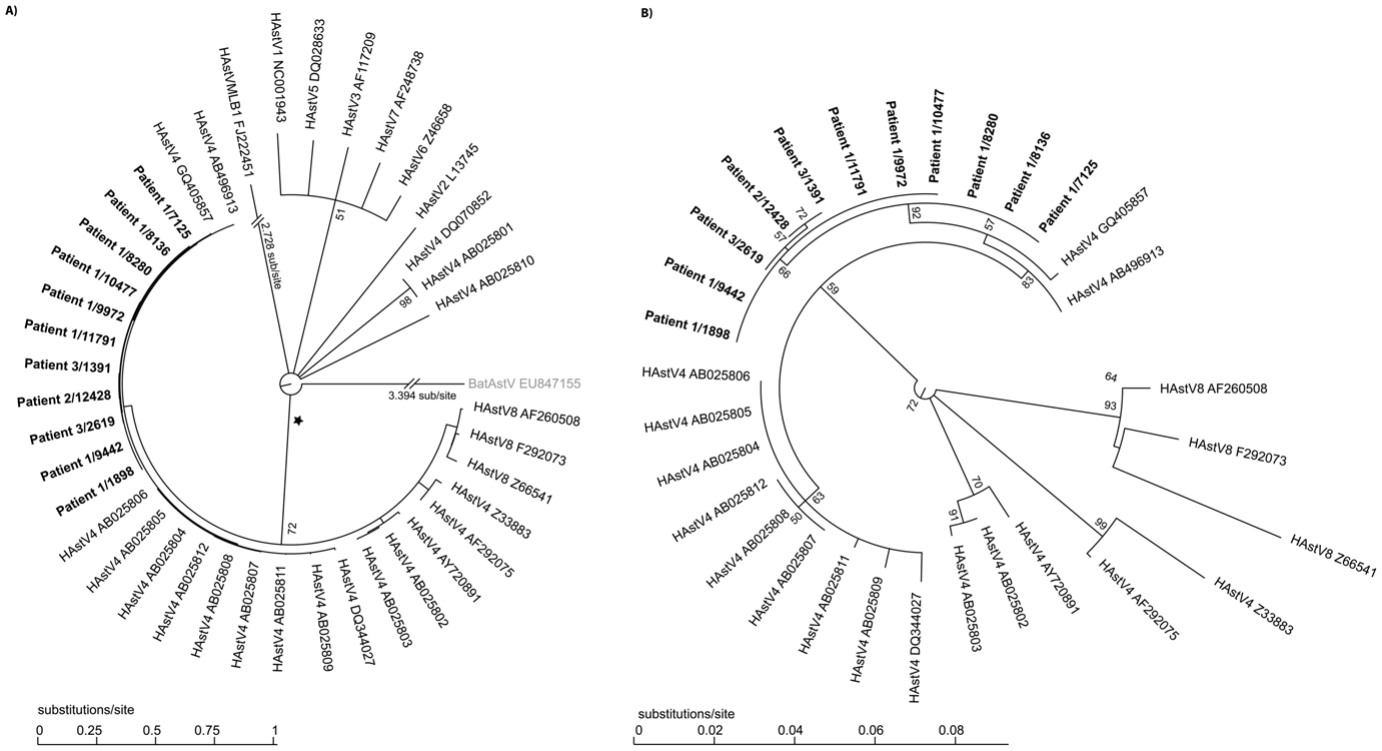
Phylogenetic analysis of the transmitted astrovirus strains. A. A maximum-likelihood-based phylogenetic tree was computed using capsid gene sequences (corresponding to nt 4325 to 4867 of the astrovirus 4 complete genome [GenBank DQ344027] or nt 1 to 543 of the capsid gene) obtained from 8 samples collected from patient 1, one from patient 2, and two from patient 3 (Table 2). Corresponding sequences from representatives of the 8 human astrovirus serotypes (HAstV) and the divergent VMLB1 human astrovirus (HastVMLB1) were included to show a comparison of evolutionary distances and relationships. Bat astrovirus (BatAstV, in light gray) was used as an outgroup. All virus strains are shown with the corresponding GenBank accession number. The numbers of substitutions per site are indicated below the tree. Because of long branches, the outgroup BatAstV, as well as the strain HAstVMLB1, are shown with annotated branch lengths. B. Blow-up of a sub-tree from panel A marked with an asterisk showing relatively short branch lengths compared to the full tree.

Stool specimens from the 3 patients were sequenced over 543 nt of the capsid gene. A phylogenetic tree revealed that the patients were infected with a unique astrovirus type 4 strain (Figure 5). Furthermore, samples collected from patient 1 96 days post-hospitalization had acquired a non synonymous change also present in patients 2 and 3 (Table 2). As expected for protracted RNA virus infections, viral populations sequenced from patient 1 between days 145 (sample no. 11196) and 250 (sample no. 1898) post-hospitalization (the period where patients 2 and 3 were infected) evolved. The exact sequence composition at the time of transmission is not known, but the sequences of sample no. 12428 (patient 2) and 1391 (patient 3) are both present in the quasispecies populations observed in sample no. 1898 (patient 1).

**Table 2:** Genetic variability of the astrovirus genome in the 3 investigated cases.

Sample N°	Days post hospitalisation of patient 1	Patient	Material	Nucleotide (amino acid)				
				*4411	*4678	*4752	*4762	*4802
7125	24	1	Stool culture	T(S)	G(G)	G(R)	T(S)	A(I)
8136	56	1	stool	T(S)	G(G)	G(R)	T(S)	A(I)
8280	62	1	stool	T(S)	G(G)	G(R)	T(S)	A(I)
9442	96	1	stool	T(S)	G(G)	G(R)	T(S)	**R(I/V)**
9972	110	1	stool	T(S)	G(G)	G(R)	T(S)	**G(V)**
10477	124	1	stool	T(S)	G(G)	G(R)	T(S)	**G(V)**
11196	145	1	stool	T(S)	*R (G)*	G(R)	T(S)	**G(V))**
1898	250	1	Stool culture	*Y(S)*	G(G)	**R (R/K)**	T(S)	**R(I/V)**
12428	#180	2	Stool culture	*C (S)*	G(G)	G(R)	T(S)	**G(V)**
1391	&237	3	stool	*C (S)*	G(G)	G(R)	T(S)	**G(V)**
2619	272	3	stool	*Y (S)*	*R (G)*	G(R)	*Y (S)*	**G(V)**

The astrovirus genome was sequenced over a 651 nt portion (corresponding to nt 4216 to 4867 of the astrovirus 4 complete genome (GenBank DQ344027) covering nt 1 to 543 of the capsid gene). Sequences are available in GenBank under accession # HQ396880 to HQ396890.

*Positions relative to astrovirus 4 genome (GenBank DQ344027). Capital letters outside brackets indicate nucleotides (A,T,G,C). Brackets indicate amino acids at their respective position. Synonymous changes are indicated in italic and non synonymous changes are indicated in bold.

# 4 days after detection of 1^st^ astrovirus positive sample in patient 2.

& time of detection of 1^st^ astrovirus positive sample in patient 3.

## Discussion

We describe 3 young children with SCID in a pediatric BMT unit who developed protracted astrovirus infection with disseminated disease, which was finally fatal in one infant. Viral infection was initially suspected, but routine diagnostic molecular investigations remained negative. Generic viral discovery tools [5], [19] were then applied and led to the identification of astrovirus. Subsequent screening by specific real-time RT-PCR confirmed the presence of astrovirus RNA in multiple body sites in at least 2 of the 3 infected cases. Retrospective screening revealed that patient 1 was already infected before transplantation, possibly even before hospitalization, and secreting large amounts of virus when patients 2 and 3 were hospitalized in the same ward. We cannot completely exclude that patients 2 and 3 were infected from another source, such as external visitors, but this seems very unlikely due to the strict isolation measures applied to HSCT recipients. The most probable scenario is that patient 1 was the source of the infection to cases 2 and 3 and the virus was transmitted by healthcare staff or the children's parents due to their close contact outside the laminar airflow cabins.

The hypothesis of nosocomial infection is further reinforced by retrospective screening of samples, which revealed that astrovirus was continuously detectable in patient 1 until immune reconstitution (Figure 1; Table S1), and capsid sequence analysis of different positive samples that confirmed the presence of a unique astrovirus type 4 strain. One unlikely hypothesis is that cases 2 and 3 were infected in the community before hospitalization with low-level replicating viruses that were not detected upon hospital admission. Of note, investigation of the donor history of patients 1 and 2 showed no signs of diarrhea one month before donation. Following the suspicion of nosocomial infection, preventive measures were taken. The hand hygiene antisepsis agent was replaced by a more virucidal product, the BMT unit was cleaned room-by-room with highly virucidal surface disinfectant, and the admission of visitors to these children was restricted to their parents only.

This sequence of events illustrates the numerous challenges facing clinicians, clinical virologists, and microbiologists caring for highly immunocompromised infants. Astrovirus real-time RT-PCR could have provided an early and simple diagnosis for these patients. However, diarrhea observed in our index case could have also been caused by many other viruses and a large panel of real-time assays targeting all possible viral agents would have been necessary. Due to the diversity of potentially involved viral agents in humans [2], as well as emerging new viruses or variants, the spectrum covered by such a panel would always be an issue. In the present investigation, classical cell culture in combination with modern generic molecular detection tools provided the diagnosis. Traditional diagnosis by cell culture has technical limitations, such as low sensitivity and existence of non-cultivable viruses, and has been progressively abandoned. In this case, cell culture was the first indication for the presence of a virus. This strongly supports the notion that it is advantageous to preserve classical viral culture in centers caring for immunocompromised hosts. Of note, negative staining EM from stool suspensions or from a supernatant from positive cell culture could also have given very fast, first indications of the presence of an unexpected virus. However this technique is not available as a routine tool in our institution. Nevertheless, microarray-based assays [3], mass spectrometry [20], ultra-deep sequencing [4], and other alternative advanced technologies will likely change the diagnostic landscape once validated for clinical use.

Astrovirus is a non-enveloped, positive-strand RNA virus that infects mammalian as well as avian hosts [21]. The virus is transmitted mainly by the oro-fecal route, circulates worldwide, and is the cause of outbreaks in the community and healthcare facilities [22]. After rotavirus, it is one of the most frequent causes of viral gastroenteritis in young children [23], [24]. The infection is characterized by a gastrointestinal disease with nausea, vomiting, diarrhoea, and sometimes fever, mostly self-limited to a few days. The inflammatory response of the gastrointestinal mucosa is often minimal [1], but there is a possible association with necrotizing enterocolitis in newborns [25].

Atypical clinical presentations, such as prolonged viral shedding, have been described in immunocompromised hosts [1], [26] and this was observed in all 3 patients reported in this study. We detected also astrovirus RNA in unexpected body sites, such as the respiratory tract, blood, bone marrow, skin, and brain. Recently, a similar investigation in a child with primary immunodeficiency and progressive encephalitis proved the ability of astrovirus to infect the brain and likely cause progressive encephalitis [4]. The authors of this study could not identify astrovirus in other sites, probably due to the retrospective nature of their investigation and a limited access to specimens. By demonstrating RNA in multiple sites, our investigation supports their observation that astrovirus can reach other organs, including the central nervous system. The ability of astrovirus to cause viremia is probably due to high and protracted replication in the gastrointestinal tract. Prolonged shedding in immunocompromised hosts has been described for other gastrointestinal RNA viruses, such as rotavirus [27], ADV [28], and norovirus [29], [30], as well as respiratory viruses [31], and highlights the need for an efficient immune response to prevent dissemination and to clear these usually self-limited infections. In our cases, prolonged shedding and deficient environmental disinfection procedures could have offered opportunities for inter-individual transmission and has to be considered as the most likely cause of the observed outbreak.

In conclusion, astrovirus needs to be considered as a potential cause of protracted disease and disseminated infection in severely immunocompromised infants with primary immunodeficiency prior to and after allogeneic HSCT.

## Supporting Information

Table S1 **Virological and immunological results obtained in samples from patient 1 (index case).**
(DOC)

Table S2 Virological and immunological results obtained in samples from patient 2.
(DOC)

Table S3 **Virological and immunological results obtained in samples from patient 3.**
(DOC)

Figure S1 **Histopathology of autopsy material of patient 2.** 1. Overview of hippocampus with necroses (arrow) and meningo-ventriculo encephalitis (H&E x 10). 2. Destructive ventriculitis consisting mainly of macrophages and granulocytes (H&E x 200). 3. Meningoencephalitis consisting of macrophages, lymphocytes, plasma cells and a few granulocytes (H&E x 50).
(TIF)
