# Correction: Astrovirus Infection in Hospitalized Infants with Severe Combined Immunodeficiency after Allogeneic Hematopoietic Stem Cell Transplantation

**DOI:** 10.1371/annotation/8371c305-a69c-4225-83ae-b0b414eca31a

**Published:** 2012-01-24

**Authors:** Werner Wunderli, Astrid Meerbach, Tayfun Guengoer, Christoph Berger, Oliver Greiner, Rosmarie Caduff, Alexandra Trkola, Walter Bossart, Daniel Gerlach, Manuel Schibler, Samuel Cordey, Thomas Alexander McKee, Sandra Van Belle, Laurent Kaiser, Caroline Tapparel

There was an error in the name of the third author. The correct name is Tayfun Güngör. The correct citation is: Wunderli W, Meerbach A, Güngör T, Berger C, Greiner O, et al. (2011) Astrovirus Infection in Hospitalized Infants with Severe Combined Immunodeficiency after Allogeneic Hematopoietic Stem Cell Transplantation. PLoS ONE 6(11): e27483. doi:10.1371/journal.pone.0027483 

