# Supplementary material for: Astrovirus Infection in Hospitalized Infants with Severe Combined Immunodeficiency after Allogeneic Hematopoietic Stem Cell Transplantation
Source: PLoS One. 2011 Nov 11;6(11):e27483. doi: 10.1371/journal.pone.0027483 (PMC3214048; doi:10.1371/journal.pone.0027483)
Supplement: Table S1 — Virological and immunological results obtained in samples from patient 1 (index case). (DOC) [file pone.0027483.s001.doc]

**Table S1.**

| **Patient 1**  **sample number** | **Date** | **Material** | **Culture** | **IF** | **Astrovirus real-time PCR (CT value)** | **Comments** |
| --- | --- | --- | --- | --- | --- | --- |
|  | **18.06.08** | **HOSPITALIZATION** | | | | |
| 6521 | 25.06.08 | BAL | NEG |  | ND | ***IgG < 0.033, IgA <0.07,*** *IgM 0.71 gr/L* |
| 6631 | 30.06.08 | STOOL | NEG |  | ND | ***CD3+ 0.0;*** *CD19+ 0.16,* ***CD16+/CD56+ 0,1*** *G/L* |
| 7107 | 11.07.08 | NPS | NEG |  | ND |  |
| §7125 | 11.07.08 | STOOL | POS | pan-entero POS | POS (19.45) |  |
| 6557 | 15.07.08 | SERUM | ND |  | POS (34.06) |  |
| 7435 | 22.07.08 | STOOL | NEG |  | ND |  |
| 7608 | 28.07.08 | NPS | POS | HPIV3 POS | ND |  |
| 7672 | 29.07.08 | STOOL | NEG |  | ND |  |
| 7812 | 04.08.08 | NPS | POS | HPIV3 POS | ND |  |
| 7814 | 04.08.08 | STOOL | POS | pan-entero POS | ND |  |
| 8033 | 11.08.08 | NPS | POS | HPIV3 POS | ND |  |
| §8136 | 13.08.08 | STOOL | NEG |  | POS (16.55) | Real-time PCR HEV NEG |
| §8280 | 19.08.08 | STOOL | NEG |  | POS (13.74) | Real-time PCR HEV NEG |
| 8645 | 28.08.08 | NPS | POS | HPIV3 POS | ND |  |
| 8733 | 01.09.08 | PS | NEG |  | ND |  |
| 9075 | 10.09.08 | NPS | POS | HPIV3 POS | ND |  |
| 9076 | 10.09.08 | STOOL | NEG |  | ND | Real-time PCR HEV NEG |
| 9248 | 16.09.08 | PS | POS | HPIV3 POS | ND |  |
| 9252 | 16.09.08 | STOOL | NEG |  | ND |  |
|  | **19.09.08** | **TRANSPLANTATION (after ATG)** | | | | |
| 9443 | 22.09.08 | NPS | POS | HPIV3 POS | ND |  |
| §9442 | 22.09.08 | STOOL | NEG |  | POS (14.01) | Real-time PCR HEV NEG |
| 9675 | 29.09.08 | NPS | POS | HPIV3 POS | ND |  |
| 9730 | 30.09.08 | STOOL | NEG |  | ND | Real-time PCR HEV NEG |
| §9972 | 06.10.08 | STOOL | NEG |  | POS (13.91) | Real-time PCR HEV NEG |
| 10189 | 13.10.08 | NPS | POS | HPIV3 POS | ND |  |
| 10231 | 13.10.08 | STOOL | NEG |  | ND |  |
| 10476 | 20.10.08 | NPS | POS | HPIV3 POS | ND |  |
| *§10477 | 20.10.08 | STOOL | NEG | Astrovirus POS | POS (14.43) | Real-time PCR HEV NEG |
| 10722 | 27.10.08 | PS | POS | HPIV3 POS | ND |  |
| 10723 | 27.10.08 | STOOL | NEG |  | ND | Real-time PCR HEV NEG |
| 10966 | 03.11.08 | NPS | POS | HPIV3 POS | ND |  |
| 11010 | 04.11.08 | STOOL | NEG |  | NEG | Real-time PCR HEV NEG |
| 11194 | 10.11.08 | NPS | POS | HPIV3 POS | ND |  |
| §11196 | 10.11.08 | STOOL | NEG |  | POS (15.03 | Real-time PCR HEV NEG |
| 11408 | 17.11.08 | NPS | POS | HPIV3 POS | ND |  |
| 11647 | 24.11.08 | NPS | POS | HPIV3 POS | ND |  |
| 11798 | 27.11.08 | STOOL | ND |  | ND | Real-time PCR norovirus NEG |
| 11878 | 01.12.08 | NPS | ND |  | POS (28.8) | Real-time PCR HRV POS  Real-time PCR AdV, FluA/B, HEV, RSV NEG |
| 12032 | 04.12.08 | NPS | ND |  | NEG | Real-time PCR HRV POS |
| 12135 | 08.12.08 | NPS | POS | HPIV3 POS | POS (34.25) | Real-time PCR HRV POS |
| **TIME OF PATIENT 2 FIRST POSITIVITY** | | | | | | |
| 12424 | 14.12.08 | NPS | POS | HPIV3 POS | ND |  |
| 12430 | 15.12.08 | PS | POS | HPIV3 POS | POS (25.58) | Real-time PCR HRV NEG |
| 12688 | 22.12.08 | NPS | POS | HPIV3 POS | ND | ***CD3+ 0.08, CD19+ 0.06, CD16+/56+ 0.06*** *G/L* |
| 12868 | 29.12.08 | PS | POS | HPIV3 POS | ND |  |
| ^EM^12869 | 29.12.08 | STOOL | POS | pan-entero POS | POS(11.3) |  |
| 16 | 31.12.08 | PS | NEG |  | POS (38.7) | Real-time PCR HEV, HSV, HRV NEG |
| 76 | 05.01.09 | NPS | NEG |  | NEG | Real-time PCR HRV NEG |
| *77 | 05.01.09 | STOOL | POS | pan-entero POS  *astrovirus POS | ND |  |
| 366 | 12.01.09 | PS | POS | HPIV3 POS | NEG | Real-time PCR HEV NEG |
| 367 | 12.01.09 | STOOL | POS | pan-entero POS | POS (12.23) | Real-time PCR HEV NEG  ***CD3+ 0.28 (0.26 gamma/deltaTCR+),***  ***CD4+CD3+ 0.02, CD8+CD3+ 0.01,***  ***CD19+ 0.05, CD16+/56+ 0.02*** *G/L* |
| 645 | 19.01.09 | PS | POS | HPIV3 POS | ND |  |
| 654 | 19.01.09 | STOOL | POS | pan-entero POS | ND |  |
| 1165 | 02.02.09 | PS | POS | HPIV3 POS | ND | ***CD3+ 0.45 (0.39 gamma-deltaTCR+),***  ***CD4+CD3+ 0.06, CD8+CD3+ 0.01,***  ***CD19+ 0.09, CD16+/56+ 0.03*** G/L |
| **TIME OF PATIENT 3 FIRST POSITIVITY** | | | | | | |
| 1476 | 10.02.09 | NPS | POS | HPIV3 POS | ND |  |
| 1475 | 10.02.09 | STOOL | POS | pan-entero POS | POS (10.5) |  |
| 1902 | 23.02.09 | PS | NEG |  | ND |  |
| ^SISPA^§1898 | 23.02.09 | STOOL | POS | pan-entero POS  *astrovirus POS | POS (10.7) | Real-time PCR HEV NEG |
| 2146 | 02.03.09 | NPS | NEG |  | ND |  |
| 2142 | 02.03.09 | STOOL | NEG |  | ND |  |
| 2386 | 09.03.09 | NPS | NEG |  | NEG | Real-time PCR HEV NEG |
| 2389 | 09.03.09 | STOOL | NEG |  | POS (36.10) | Real-time PCR HEV NEG  ***CD3+ 0.64 (0.33 gamma/deltaTCR+),***  ***CD4+CD3+ 0.52, CD8+CD3+ 0.13,***  ***CD19+ 0.01, CD16+/56+ 0.07*** G/L |
| 2618 | 16.03.09 | NPS | NEG |  | ND |  |
| 2620 | 16.03.09 | STOOL | NEG |  | ND |  |
| 4756 | 14.05.09 | STOOL | NEG |  | NEG | Real-time PCR HEV NEG  *CD3+ 2.0 (alpha/betaTCR+), CD4+CD8+ 1.35*  *CD8+CD3+ 0.4,* ***CD19+ 0.15,*** ***CD16+/56+ 0.03*** *G/L* |

IF, immunofluorescence; ND, not done; POS, positive; NEG, negative; BAL, bronchoalveolar lavage; NPS, nasopharyngeal secretion; PS, pharyngeal swab; pan-enterovirus detection kit; HPIV3, human parainfluenza 3 virus; HEV,human enterovirus; HRV, human rhinovirus; RSV, respiratory syncytial virus; AdV, adenovirus; Flu, influenza virus; HSV, herpes simplex virus; § sample sequenced, ^EM^sample analyzed by electron microscopy, ^SISPA^ sample used for SISPA, * samples retrospectively tested by immunofluorescence with an anti-astrovirus monoclonal antibody. Italic: Lymphocyte subsets measured by flow cytometry (FACS): CD3+= T-cells; CD4+CD3+ = CD4+ Helper T-cells; CD8+CD3+= CD8+ cytotoxic T-cells; CD19= B-cells; CD16+/56+= NK-cells; IgG, IgM, IgA= serum immunoglobulins (measured by nephelometry); G/L= Giga/liter; gr/L= gram/liter; TCR+= T-cell receptor. Pathological values are indicated in bold characters.
