# Supplementary material for: Astrovirus Infection in Hospitalized Infants with Severe Combined Immunodeficiency after Allogeneic Hematopoietic Stem Cell Transplantation
Source: PLoS One. 2011 Nov 11;6(11):e27483. doi: 10.1371/journal.pone.0027483 (PMC3214048; doi:10.1371/journal.pone.0027483)
Supplement: Table S2 — Virological and immunological results obtained in samples from patient 2. (DOC) [file pone.0027483.s002.doc]

**Table S2.**

| **Patient 2**  **sample number** | **Date** | **Material** | **Culture** | **IF** | **Astrovirus- PCR (CT value)** | **Comments** |
| --- | --- | --- | --- | --- | --- | --- |
| 11783 | 26.11.08 | PS | NEG |  | ND | *IgG 6.1,* ***IgA <0.07, IgM 0.18 gr/L***  ***CD3+ 0.0****, CD19+ 1.7, CD16+/56+ 0.31 G/L* |
|  | **28.11.08** | **HOSPITALIZATION** | | | | |
| 11847 | 29.11.08 | PS | NEG |  | ND |  |
| 11848 | 29.11.08 | STOOL | NEG |  | ND |  |
|  | **05.12.08** | **TRANSPLANTATION (after ATG, targeted Busulfan)** | | | | |
| 12134 | 08.12.34 | NS | NEG |  | ND |  |
| 12288 | 11.12.08 | plasma |  |  | POS (35.86) | Real-time PCR AdV, HEV, HSV-1,2,  VZV NEG |
| 12296 | 11.12.08 | STOOL | POS | pan-entero POS | POS (13.77) | Real-time PCR HEV NEG  ***CD3+ 0.0, CD19+ 0.11, CD16+/56+ 0.0 G/L*** |
| §12428 | 15.12.08 | STOOL | POS | pan-entero POS | POS (10.65) | Real-time PCR HEV NEG |
| 12429 | 15.12.08 | PS | NEG |  | NEG | Real-time PCR HEV NEG |
| 12472 | 16.12.08 | plasma |  |  | POS (32.01) | Real-time PCR parvovirus, HEV NEG |
| 12538 | 17.12.08 | urine | NEG | CMV-EA NEG | ND | ***CD3+ 0.0, CD19+ 0.11, CD16+/56+ 0.03 G/L*** |
| 12641 | 19.12.08 | plasma |  |  | POS (34.73) | Real-time PCR HEV NEG  ***CD3+ 0.0, CD19+ 0.07, CD16+/56+ 0.08 G/L*** |
| 12665 | 20.12.08 | skin swab | NEG |  | ND |  |
| 12666 | 21.12.08 | PS | NEG |  | NEG | Real-time PCR HEV, HRV NEG |
| 12667 | 21.12.08 | tracheal secretion | NEG |  | NEG | Real-time PCR HEV, HRV, NEG |
| 12668 | 21.12.08 | vesicle swab | NEG |  | POS (38.47) | Real-time PCR CMV, VZV, HEV NEG |
| 12738 | 22.12.08 | CSF | NEG |  | NEG | Real-time PCR HEV NEG |
| A08 424-01 | PM | brain |  |  | POS (32.27/33.37) | Real-time PCR in duplicate |
| A08 424-20 | PM | heart |  |  | POS (undet/38.73) | Real-time PCR in duplicate |
| A08 424-31 | PM | lung |  |  | POS (undet/undet/38.44) | Real-time PCR in triplicate |
| A08 424-41 | PM | liver |  |  | NEG |  |
| A08 424-51 | PM | spleen |  |  | POS (undet/37.98) | Real-time PCR in duplicate |
| A08 424-53 | PM | bone/bone marrow |  |  | POS (29.30/30.62) | Real-time PCR in duplicate |
| A08 424-65 | PM | kidney |  |  | POS (36.83/37.11) | Real-time PCR in duplicate |
| A08 424-45 | PM | intestine |  |  | POS (22.25) |  |

IF, immunofluorescence; ND, not done; POS, positive; NEG, negative; PS, pharyngeal swab; NS, nasal swab; CSF, cerebrospinal fluid; undet, undetectable; pan-entero; pan-enterovirus detection kit; HEV,human enterovirus; HRV, human rhinovirus; AdV, adenovirus; HSV, herpes simplex virus; VZV, varicella zoster virus; CMV, cytomegalovirus; CMV-EA, cytomegalovirus early antigen.§ sample sequenced. Italic: Lymphocyte subsets measured by flow cytometry (FACS): CD3+= T-cells; CD4+CD3+ = CD4+ Helper T-cells; CD8+CD3+= CD8+ cytotoxic T-cells; CD19= B-cells; CD16+/56+= NK-cells; IgG, IgM, IgA= serum immunoglobulins (measured by nephelometry); G/L= Giga/liter; gr/L= gram/liter; TCR+= T-cell receptor. Pathological values are indicated in bold characters.
