# Supplementary material for: Astrovirus Infection in Hospitalized Infants with Severe Combined Immunodeficiency after Allogeneic Hematopoietic Stem Cell Transplantation
Source: PLoS One. 2011 Nov 11;6(11):e27483. doi: 10.1371/journal.pone.0027483 (PMC3214048; doi:10.1371/journal.pone.0027483)
Supplement: Table S3 — Virological and immunological results obtained in samples from patient 3. (DOC) [file pone.0027483.s003.doc]

**Table S3.** Virological and immunological results obtained in samples from patient 3.

| Patient 3  sample number | Date | Material | Culture | IF | Astrovirus- PCR (CT value) | Comments |
| --- | --- | --- | --- | --- | --- | --- |
|  | **12.01.09** | **HOSPITALIZATION** | | | | |
| 415 | 13.01.09 | NPS | NEG |  | ND | *CD3+ 2.4, CD4+CD3+ 2.11,* ***CD8+CD3+ 0.21****,*  *CD19+ 2.1, CD16+/56+ 0.34 G/L* |
| 449 | 13.01.09 | STOOL | NEG |  | ND |  |
| 485 | 14.01.09 | PS | NEG |  | ND |  |
| 921 | 16.01.09 | PS | NEG |  | ND |  |
|  | **23.01.09** | **TRANSPLANTATION (after Alemtuzumab, Fludarabin and targeted Busulfan)** | | | | |
| 922 | 26.01.09 | STOOL | NEG |  | ND |  |
| 1164 | 02.02.09 | NPS | NEG |  | ND |  |
| 1214 | 03.02.09 | STOOL | NEG |  | ND |  |
| 1426 | 09.02.09 | NPS | NEG |  | ND |  |
| *§1391 | 09.02.09 | STOOL | POS | pan-entero POS  astrovirus POS | POS (9.73) | Real-time PCR norovirus NEG |
| 1474 | 10.02.09 | STOOL | POS | pan-entero POS | ND | rotavirus antigen, AdV, norovirus NEG, |
| 1600 | 12.02.09 | STOOL |  |  | ND | rotavirus antigen, AdV NEG,  Real-time PCR norovirus NEG |
| 1678 | 16.02.09 | PS | NEG |  | ND |  |
| 1679 | 16.02.09 | STOOL | POS | pan-entero POS | ND |  |
| 1818 | 19.02.09 | STOOL | POS | pan-entero POS | POS (9.6) | Real-time PCR HEV NEG |
| 1899 | 23.02.09 | PS | NEG |  | POS (34.05) | Real-time PCR HEV NEG |
| 1965 | 24.02.09 | STOOL | POS | pan-entero POS | ND | Real-time PCR HEV NEG |
| 2144 | 02.03.09 | NPS | NEG |  | NEG | Real-time PCR HEV NEG |
| 2130 | 02.03.09 | STOOL | POS | pan-entero POS | POS (10.52) | Real-time PCR HEV, AdV NEG  ***CD3+ 0.02, CD19+ 0.03,*** CD16+/56+ 0.13 G/L |
| 2387 | 09.03.09 | NPS | NEG |  | POS (35.06) | Real-time PCR HEV NEG |
| 2388 | 09.03.09 | STOOL | POS | pan-entero POS | ND | Real-time PCR HEV NEG |
| 2615 | 16.03.09 | PS | NEG |  | ND |  |
| §2619 | 16.03.09 | STOOL | POS | pan-entero POS | POS (19.7) | ***CD3+ 0.19, CD4+CD3+ 0.14, CD8+CD3+ 0.05,***  ***CD19+ 0.29,*** CD16+/56+ 0.28 G/L |
| 3153 | 01.04.09 | NPS | NEG |  | ND |  |
| 3154 | 01.04.09 | STOOL | POS | pan-entero POS | POS (18.24) |  |
| 9235 | 09.07.09 | STOOL | NEG |  | ND | ***CD3+ 0.38, CD4+CD3+ 0.22, CD8+CD3+ 0.07,***  ***CD19+ 0.22,*** CD16+/56+ 0.33 G/L |

IF, immunofluorescence; ND, not done; POS, positive; NEG, negative; NPS, nasopharyngeal secretion; PS, pharyngeal swab; pan-entero; pan-enterovirus detection kit; HEV,human enterovirus; AdV, adenovirus; § sample sequenced, * samples retrospectively tested by immunofluorescence with an anti-astrovirus monoclonal antibody. Italic: Lymphocyte subsets measured by flow cytometry (FACS): CD3+= T-cells; CD4+CD3+ = CD4+ Helper T-cells; CD8+CD3+= CD8+ cytotoxic T-cells; CD19= B-cells; CD16+/56+= NK-cells; G/L= Giga/liter. Pathological values are indicated in bold characters.
