# Supplementary figures and images for: Astrovirus Infection in Hospitalized Infants with Severe Combined Immunodeficiency after Allogeneic Hematopoietic Stem Cell Transplantation
Source: PLoS One. 2011 Nov 11;6(11):e27483. doi: 10.1371/journal.pone.0027483 (PMC3214048; doi:10.1371/journal.pone.0027483)

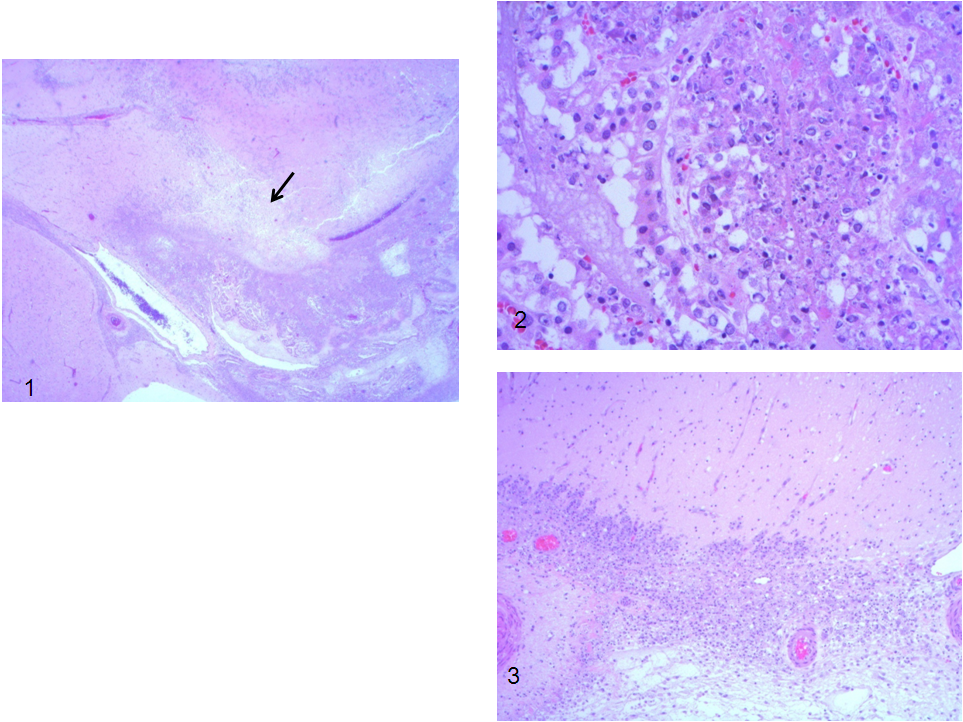

Supplement: Figure S1 — Histopathology of autopsy material of patient 2. 1. Overview of hippocampus with necroses (arrow) and meningo-ventriculo encephalitis (H&E x 10). 2. Destructive ventriculitis consisting mainly of macrophages and granulocytes (H&E x 200). 3. Meningoencephalitis consisting of macrophages, lymphocytes, plasma cells and a few granulocytes (H&E x 50). (TIF) [file pone.0027483.s004.tif]
